# Factors of significance for the ability of fighter pilots to visually indicate the magnitude of roll tilt during simulated turns in a centrifuge

**DOI:** 10.1177/03010066231209847

**Published:** 2023-11-09

**Authors:** Andreas Brink, Michail E. Keramidas, Arne Tribukait, Ola Eiken

**Affiliations:** Division of Environmental Physiology, Swedish Aerospace Physiology Center, 7655 KTH Royal Institute of Technology, Stockholm, Sweden; Department of Clinical Neuroscience, Section for Eye and Vision, 27106 Karolinska Institute, Stockholm, Sweden; Division of Environmental Physiology, Swedish Aerospace Physiology Center, KTH Royal Institute of Technology, Stockholm, Sweden

**Keywords:** aviation, spatial disorientation, spatial orientation, subjective visual horizon, vestibular psychophysics

## Abstract

During coordinated flight and centrifugation, pilots show interindividual variability in perceived roll tilt. The study explored how this variability is related to perceptual and cognitive functions. Twelve pilots underwent three 6-min centrifugations on two occasions (G levels: 1.1G, 1.8G, and 2.5G; gondola tilts: 25°, 56°, and 66°). The subjective visual horizontal (SVH) was measured with an adjustable luminous line and the pilots gave estimates of experienced G level. Afterward, they were interrogated regarding the relationship between G level and roll tilt and adjusted the line to numerically mentioned angles. Generally, the roll tilt during centrifugation was underestimated, and there was a large interindividual variability. Both knowledge on the relationship between G level and bank angle, and ability to adjust the line according to given angles contributed to the prediction of SVH in a multiple regression model. However, in most cases, SVH was substantial smaller than predictions based on specific abilities.

Spatial disorientation is common in aviation because visual references are often missing (as when flying in clouds or darkness), and since during complex motion patterns—or changes in the gravitoinertial force field (G vector; vectorial sum of the inertial forces and the force of gravity)—the sense of balance tends to provide erroneous or conflicting information on angular movements or the direction of gravity. Although pilots are taught to rely on the flight instruments, rather than their own bodily sensation of position, attitude, and speed, spatial disorientation remains a major cause or contributory factor in many flight accidents (Gibb et al., 2011).

A basic flight maneuver is the coordinated turn. When entering a turn in a typical manner, the semicircular canals sense the roll-angular velocity, and the resulting signal is integrated over time at central nervous level to an estimate of undergone angular displacement (Glasauer, 1992). However, since the resultant G vector does not change direction with respect to the pilot, the message from the otolith organs is that the head remains in the upright position. This intrasensory conflict can be induced also in a large centrifuge with a tangentially pivoted gondola. In spite of certain physical differences between the real aircraft and centrifugation (i.e., during a real aircraft turn, the pilot typically maintains constant speed while reducing the radius of the aircraft's trajectory, whereas in the centrifuge the turn commences with a tangential acceleration and has a fixed radius, typically smaller than 10 m), the perception of roll tilts is very similar in the two systems, in pilots (Tribukait et al., 2023) and in nonpilots (Tribukait et al., 2016).

During gondola centrifugation and coordinated flight, there is generally a considerable underestimation of the physical roll tilt. Thus, in participants without flight experience, the indicated tilt angle averages 30%–40% of the real tilt (Tribukait & Eiken, 2006). Further, during constant angular velocity of the centrifuge, the indicated tilt declines exponentially with time, approximating 0° within a few minutes. Conspicuously, when pilots are exposed to a prolonged turn in a centrifuge or aircraft, a majority of them indicate a perception of roll tilt that does not decline with time; they often report verbally a sensation of being in a sustained coordinated turn (Tribukait et al., 2011, 2023). In addition, although there is a large interindividual variability in the magnitude of indicated roll tilt, the responses are, in general, larger in pilots (50%–65% of the real tilt) than in nonpilots (Tribukait et al., 2011).

These findings give rise to two questions. Firstly, why do pilots commonly maintain a constant perception of roll tilt during a prolonged turn or centrifuge run? Secondly, why are only a minority of the pilots able to correctly indicate the magnitude of roll tilt? One possibility is that pilots differ in their capability on using the semicircular canal input elicited by entering a turn (or accelerating the centrifuge). This canal-based sensation of roll-angular displacement might, irrespective of its magnitude, be maintained by a short-term memory acquired via flight experience. Alternatively, the initial impression and visual indication of the tilt angle could be stored in a visual working memory. In the latter case, the persistence of indicated roll tilt would be susceptible to visual distraction tasks. Another possibility, suggested in earlier studies (Tribukait et al., 2011, 2023), is that pilots tend to estimate, consciously or subconsciously, the magnitude of the G vector and, on the basis of knowledge on the relationship between G load and bank angle, “translate” the sensation of increased weight into an estimate of bank angle. If so, visual distraction tasks would not result in a reduced magnitude on indicated roll.

Accordingly, the general aim of the present study was to examine the ability of pilots to estimate, using reason and flight experience, the roll tilt angle during simulated coordinated turns in a centrifuge. This is in contrast to earlier studies in which pilots were instructed not to reason but to adjust the line according to the intuitive imagination of the external horizon (cf. Tribukait et al., 2011, 2023). Specifically, we wanted to elucidate how the pilots’ indications of perceived roll tilt are dependent on their estimates of G load (e.g., via somatosensory impressions) and whether such estimates in combination with (a) knowledge of the relationship between G levels and bank angle, and (b) the ability to adjust the line to slants, numerically mentioned by the experimenter, correspond to the indications actually obtained. A secondary aim was to establish whether the pilots’ ability to maintain the initial sensation of roll tilt is sensitive to a visual distraction task.

## Materials and Methods

### Participants

Twelve male fighter pilots, aged 29–49 (*M* = 42) years, with flight experience of 1,400–7,000 h (*M* = 2,900 h), participated. All were recruited from the Malmen Air Force Base, Linköping (Sweden). No women took part, because, at the time, there were no active female fighter pilots in the Swedish Air Force. Before giving their written consent to participate, the participants were informed in detail about the experimental procedures and that they could terminate their participation at any time. The experimental protocol and test procedure were in accordance with the declaration of Helsinki and were approved by the Human Ethics Committee of Stockholm (Ref no: 2016/535-31).

### Equipment

#### Centrifuge

**Figure 1.**
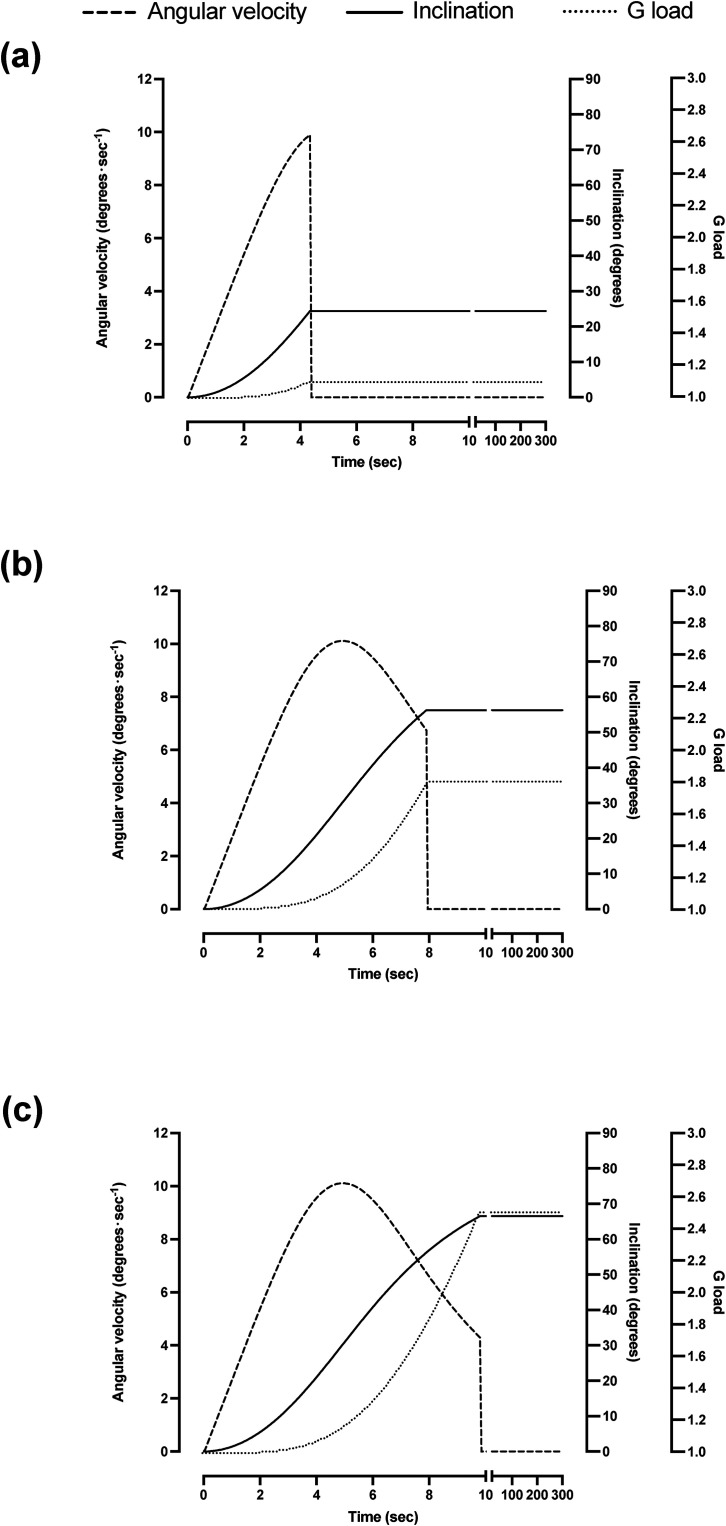
Roll-angular velocity, gondola inclination, and resultant +Gz force as functions of time during acceleration of the centrifuge from 1 g to 1.1 G (a), 1.8 G (b), and 2.5 G (c).

The experiments were performed in the Dynamic Flight Simulator (Wyle Laboratories, Inc., El Segundo, CA, USA) at the Flight Physiological Centre in Linköping. The radius of the centrifuge is 9.1 m, and as seen from above, it rotates counterclockwise. The vertical axis of the gondola, hence the long axis (*z*-axis) of the pilot, was aligned with the resultant gravitoinertial force vector, throughout each centrifuge trial, including during the roll-angular displacement phases upon acceleration and deceleration of the centrifuge (Figure 1). Planetary acceleration and deceleration were 7.8°·s^−2^. The mean roll-plane angular velocity was approximately 6°·s^−1^, i.e., well above the stimulus threshold for the semicircular canals (cf. Allred & Clark, 2023; Lim et al., 2017).

Facing forward, the pilot was fixed in the gondola seat by safety belts. A head holder ensured that the interocular line was maintained perpendicular to the gravitoinertial force vector. In addition, the head was adjusted in the sagittal plane (pitch) so that a line from the external auditory meatus to the inferior margin of the orbit was tilted about 10° (anterior end up). The head holder also served to minimize movement-induced Coriolis effects. During the experiments, the gondola was completely darkened. The pilot was observed in infrared light by a video camera, and he always had the possibility to communicate with the experimenter by means of a two-way intercom system. The pilot's heart rate and rhythm were monitored continuously by electrocardiography.

#### Subjective Visual Horizontal Measurements

In front of the pilot, at a straight-ahead eye-level position 80 cm from the pilot's eyes, there was a line (75 mm long and 1.7 mm wide) of red light-emitting diodes. The line was mounted on the axle of a digital servo (DSR 1015, Thunder Tiger Corp., Taichung City, Taiwan). Its axis of rotation coincided with the pilot's naso-occipital (visual) axis. The servo was controlled by a microprocessor (Arduino UNO) via a custom-made computer program (LabView, National Instruments Corporation, Austin, TX, USA). The pilot used two push buttons on a remote control to adjust the line, every time it was switched on, so that it appeared to be horizontal [i.e., corresponded with the pilot's imagination of the horizon of the external world; subjective visual horizontal (SVH)]. If the pilot kept one of these buttons pressed, the rotation of the line was 11°·s^−1^; by briefly tapping the buttons, the pilot could adjust the orientation of the line in steps of 0.2°. When pleased with a setting, the pilot pressed a third button, which extinguished the line. The deviation of the line from the gravitoinertial horizontal was automatically recorded with an accuracy of 0.1°. The line was then instantaneously offset 8°–26° (randomly), alternately clockwise and counterclockwise with respect to the pilot's latest setting, and it was switched on again after a latency period of 1 s. The pilots typically made 10–15 settings of the line per minute. Data were continuously stored on the computer and were subsequently analyzed using LabView-based software.

### Experimental Protocol

All pilots participated in two experimental sessions, separated by approximately 6 months. Each session consisted of three centrifuge trials, each lasting 6 min and being preceded and followed by, respectively, a 2-min and 20-s recording of SVH at 1 g. The centrifuge was accelerated from stationary to +Gz plateaus of 1.1 G, 1.8 G, and 2.5 G. The order of the trials was alternated among pilots but was the same for each pilot in the two sessions. The trials were performed with a 5-min rest period between them, which should be sufficient to avoid “carry-over” effects on the SVH determinations (cf. Tribukait & Eiken, 2005b). The pilot, who remained in the gondola throughout each session, was informed that he would be exposed to G loads ≤  + 4 Gz, but he was unaware of the G level and the order of the trials. The pilot was instructed to set the line so that it coincided with his SVH (see previous paragraph), using all available information, including his sense of tilt as well as of the G load, and his knowledge of the relation between bank angle and G load. Early during each centrifugation (between 40 and 80 s after reaching the G plateau), the pilot was also asked to give a verbal assessment of the G load encountered and to briefly describe how he reasoned about the settings of the line. The latter was done to ensure that the pilot was following the instructions and indicating SVH (i.e., using an allocentric frame of reference) and not setting the line parallel to the floor of the gondola [subjective transversal plane of the head, STP, i.e., using an egocentric frame of reference (Tribukait & Eiken, 2005a)]; the pilot continued his SVH settings during this interrogation. After each trial, the pilot was asked if he had experienced the G load during the trial as constant or varying and whether the impression reminded him of any flight maneuver.

In session 1, SVH settings were executed and monitored throughout each 6-min G plateau, whereas in session 2, the setting/monitoring of SVH was interrupted during 40 s of the G plateau. Thus, session 2 comprised three additional tasks. Firstly, at min 4 of each 6-min G plateau (i.e., at 1.1 G, 1.8 G, and 2.5 G), the pilot was instructed to align the luminous line so that it would be parallel with the gondola floor (i.e., STP). After 40 s of STP settings (henceforth termed the distraction phase), the pilot was requested to return to the task of indicating SVH for the remaining 1:20 min. Secondly, after the completion of all G trials, the pilot remained seated in the gondola in the static 1-g environment for ∼10 min and was then requested to, by using the luminous line, indicate 22 numerically presented angles, 11 on the right and 11 on the left, ranging from 10° to 80° (i.e., left and right, 10°, 15°, 20°, 30°, 40°, 45°, 50°, 60°, 70°, 75°, and 80°); this procedure was performed twice, and an average value was calculated for each angle (based on two settings on the right and two on the left). We defined the two angles closest to the horizontal (10°) and vertical (80°), respectively, as “near cardinal” and the others (15°–75°) as “oblique.” Thirdly, after debarking from the centrifuge, each pilot was interrogated about his knowledge of the relationship between bank angle and +Gz load during level turn. Thus, the experimenter numerically mentioned either G levels (range: 1.1–7.0 G), and the pilot was asked for the corresponding bank angles (subtask 1) or bank angles (10°–80°), and the pilot was asked for the corresponding G level (subtask 2); the two trials were performed in a counterbalanced order. In the second and third tasks, the sequence of the presented angles and G levels was pseudorandomized between participants.

### Data and Statistical Analyses

Tilt of SVH to the right (right end of the line set down, from the pilot's point of view) was denoted positive; tilt to the left was denoted negative. SVH was monitored continuously throughout each 6-min G trial (aside from the 40-s distraction phase in session 2 trials); yet, our analysis was based on the SVH values obtained during minutes 0–1 (initial phase) and 3–4 (final phase, i.e., prior to the distraction phase in session 2). Thus, based on results from our previous studies (Tribukait et al., 2011), it was presumed that any major and rapid changes in SVH would occur early during the G plateau. Therefore, the initial SVH tilt, at time zero, was obtained by linear curve fitting to the SVH values of the first minute, with the intercept of the regression line being defined as the “initial” SVH. As regards the final value of the SVH, we assumed that the SVH had stabilized within 3 min at the G plateau (Tribukait & Eiken, 2006). Thus, the final value of SVH was calculated as the mean of the data points obtained during minutes 3–4, prior to the distraction phase. The STP values obtained during the 40-s distraction phase were averaged. To evaluate the time course of the postdistraction response, values were divided into two periods: (a) from 0 to 30 s (*post-1* distraction) and (b) from 30 to 80 s (*post-2* distraction). Assessment of each pilot's ability to indicate numerically presented roll tilt angles corresponding to those of the three trials (25°, 56°, and 66°) was achieved by interpolation between settings at adjacent angles.

Data are presented as means (standard deviation). A two-way [session × time point (initial × final)] and one-way [time point (before distraction × post-1 distraction × post-2 distraction)] repeated-measures analysis of variance (ANOVA) was employed to evaluate any inter- and intrasession differences in SVH, respectively. Mauchly's test was conducted to assess the sphericity, and if necessary, the Greenhouse–Geiser *ɛ* correction was used to adjust the degrees of freedom. When ANOVAs revealed significant effects, multiple pairwise comparisons were performed with Tukey's honestly significant difference post hoc test. Paired sample Student's *t*-test was used to assess the between-session variations in the perception of +Gz load and the deviation changes in the adjustment of bank angles. The comparison of two standard deviations was performed by means of the *F*-test, when necessary.

Data from all G trials from both sessions were pooled to define a wide range [i.e., 12 participants × 3 G trials (1.1 G, 1.8 G, and 2.5 G) × 2 sessions = 72 observations]. The Pearson product–moment correlation was used to evaluate the relation between SVH (i.e., the mean of the initial and final SVH obtained during each trial/session) and (a) the +Gz load perception (i.e., the G value provided by each participant in the corresponding trial/session), (b) the knowledge of the relationship between bank angle and +Gz load, and (c) the capacity to indicate the angle in a 1-g environment. Considering that the knowledge of relation between G and angle, and the capacity to indicate angle, was not assessed at the specific tested tilt (bank) angles (i.e., at 25°, 56°, and 66°), they were interpolated from the individual values obtained during the respective task. Magnitudes of correlations were interpreted qualitatively using Cohen's scale: *r* < .1: trivial, .1–.3: small, .3–.5: moderate, and >.5: large. A multiple linear regression analysis was also performed, wherein the independent variables were all three capacities, and the dependent variable was SVH. Multicollinearity was evaluated by a variance inflation factor (VIF), which was always <5; for the +Gz load perception: VIF = 2.6, for the knowledge of the relationship between bank angle and +Gz load: VIF = 4.0, and for the capacity to adjust the angle in a 1-g environment: VIF = 4.9. The relative contribution of each independent variable (i.e., capacity) was calculated by the absolute value of β coefficient for each capacity derived from the multiple linear regression model. Thus, the percentage contribution of a given capacity was estimated from the β coefficient of this capacity as a function of the sum of β coefficients of all capacities.

Statistical analyses were performed using Statistica 8.0 (StatSoft, Tulsa, OK, USA) and Prism 9.3 (GraphPad Software Inc., San Diego, CA, USA). The α-level of significance was set a priori at 0.05.

## Results

All trials were performed without any adverse events. None of the participants showed any signs of motion sickness or felt uneasy during any trial.

### SVH Measurements during the G Trials

At 1 g, SVH was close to the true horizon both before [session 1: −2 (2)°, session 2: −2 (1)°; *t* = (11), *p* = .83] and immediately after [session 1: −2 (2)°, session 2: −2 (1)°; *t* = (11), *p* = .84] centrifugation. There was also no orderly difference between the 1-g SVH values [in session 1: trial 1: −2 (2)°, trial 2: −2 (2)°, and trial 3: −2 (2)° and in session 2: trial 1: −2 (1)°, trial 2: −2 (2)°, and trial 3: −2 (2)°; *p* = .17]. In the beginning of the G plateau, all participants reported that they experienced a sensation of roll tilt, which was also confirmed by their SVH indications (Table 1). With one exception, the participants perceived the G load as constant during the course of each high G exposure. All participants reported that the G trials reminded them of prolonged coordinated level turns. Since the gondola was tilted to the left, most pilots indicated SVH to the right, as they were instructed to set the imaginary horizon. Two participants, however, made STP settings during the first session. After complementary instructions, both repeated the session, during which they were able to adequately indicate SVH (the initial run, during which they indicated STP, was not included in the analysis).

**Table 1.** Initial and final SVH values for each G load. Individual and group means (SD) are presented. Gondola inclinations were 25° (1.1 G), 56° (1.8 G), and 66° (2.5 G).

	1.1 G				1.8 G				2.5 G			
	Session 1		Session 2		Session 1		Session 2		Session 1		Session 2	
Subject	Initial	Final	Initial	Final	Initial	Final	Initial	Final	Initial	Final	Initial	Final
A	2	1	10	4	14	3	18	10	16	5	25	33
B	11	9	28	10	34	40	43	43	34	53	38	74
C	21	−4	18	22	9	12	46	53	39	50	64	65
D	34	24	31	16	22	40	48	53	66	61	63	71
E	23	8	24	1	23	20	27	30	39	36	37	45
F	15	15	29	43	24	26	39	55	34	33	44	57
G	11	3	15	23	46	34	44	54	38	52	58	51
H	30	44	30	40	65	44	37	53	46	50	51	52
I	7	4	6	4	6	15	20	17	13	30	19	42
J	13	0	8	−2	14	17	3	−1	37	57	2	6
K	27	10	24	−3	51	58	52	39	55	67	56	58
L	25	30	29	43	52	53	34	51	42	57	55	66
*m*	18	12	21	17	30	30	34	38	38	46	43	52^a^
*SD*	10	14	9	17	19	17	15	20	14	17	19	19

*Note.* Sessions 1 and 2 were separated by a 6-month period. Initial SVH: SVH values obtained at time zero (see also under methods) during the first minute of each G plateau. Final SVH: average SVH values obtained during the minutes 3–4 of each G plateau.

^a^ Significantly different from the initial SVH; *p* < .05.

Table 1 summarizes the mean and individual SVH values during each centrifugation trial. SVH did not differ between sessions at any time point [1.1 G: *F*(1, 11) = 0.28, *p* = .60; 1.8 G: *F*(1, 11) = 0.59, *p* = .45; and 2.5 G: *F*(1, 11) = 0.11, *p* = .74]. SVH was relatively stable over the course of the 1.1-G [*F*(1, 11) = 2.76, *p* = .12] and 1.8-G [*F*(1, 11) = 1.33, *p* = .27] trials. During the 2.5-G trials, by contrast, SVH increased over time [*F*(1, 11) = 11.60, *p* = .005], by 9° in the second session (*p* = .03), and with a similar tendency (8° increase) albeit not statistically significant in the first session (*p* = .07). Overall, the G load was overestimated by 20%–30% (Table 2). Yet, no inter-session differences were detected as regards the perceived G load, either at 1.1 G [*t*(11) = 2.01, *p* = .07], at 1.8 G [*t*(11) = 0.11, *p* = .91], or at 2.5 G [*t*(11) = 1.12, *p* = .28].

**Table 2.** Reported estimates of G level during centrifugation at 1.1 G, 1.8 G, and 2.5 G. Individual values and group means (SD) are shown.

	1.1 G		1.8 G		2.5 G	
Subject	Session 1	Session 2	Session 1	Session 2	Session 1	Session 2
A	2.5	1.8	2.5	2.5	3.3	3.0
B	1.2	1.1	1.8	1.6	3.0	4.5
C	1.5	1.5	1.8	2.5	3.0	3.5
D	1.5	1.1	2.3	2.0	3.0	3.0
E	1.5	1.3	2.0	3.0	2.5	4.5
F	1.5	1.2	2.0	2.0	2.8	2.8
G	1.5	1.5	2.5	2.5	3.0	3.0
H	2.0	1.5	2.0	2.0	3.5	4.0
I	1.3	1.3	2.2	2.0	3.0	4.0
J	1.3	1.1	2.0	1.1	3.3	2.3
K	1.1	1.5	2.0	1.5	3.0	2.0
L	1.3	1.3	2.3	2.5	3.0	3.3
*m*	1.5	1.4	2.1	2.1	3.0	3.3
*SD*	0.4	0.2	0.2	0.5	0.3	0.8

*Note.* Sessions 1 and 2 were separated by a 6-month period. *p* > .05.

**Figure 2.**
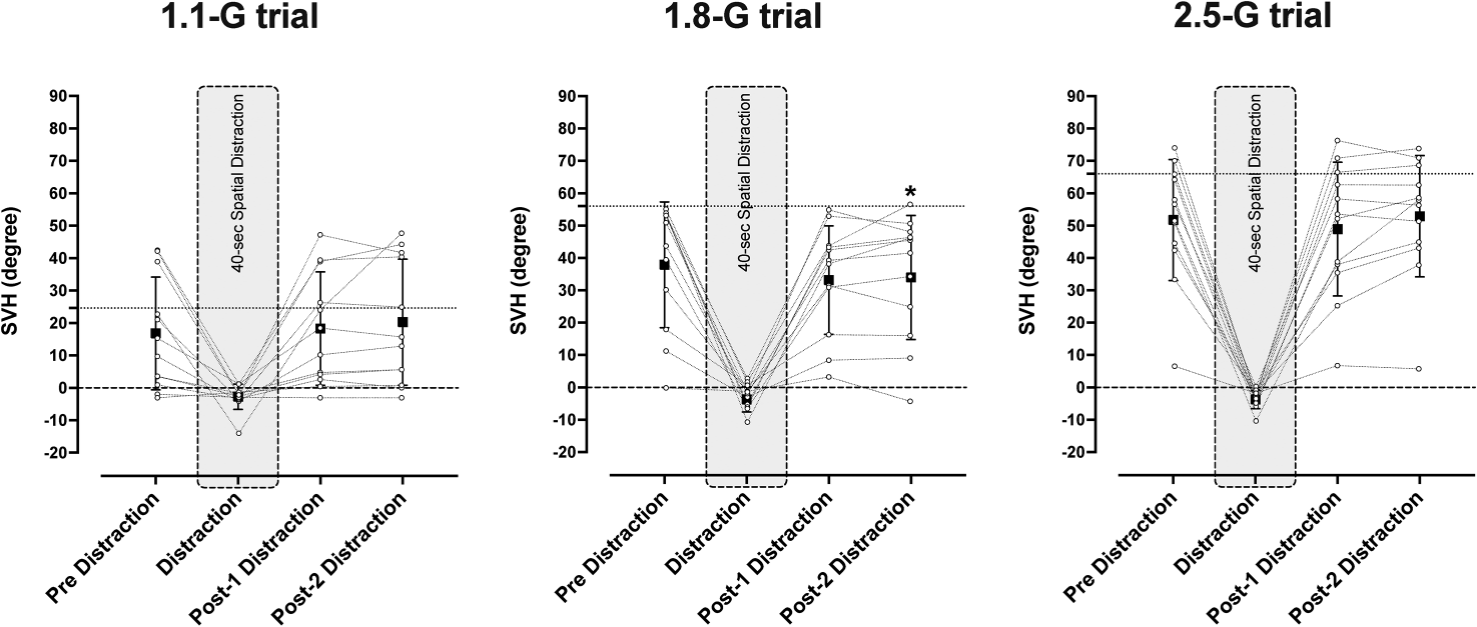
Mean (standard deviation) and individual values of subjective visual horizontal (SVH) measurements obtained before and after (post-1: 0–30 s, post-2: 30–80 s) a 40-s distraction phase (shaded area) during sustained exposure to 1.1 G, 1.8 G, and 2.5 G in session 2. The dotted line indicates the true gondola inclination. During the distraction phase, the pilots indicated their subjective transversal plane. Dashed line indicates the true transversal plane (0°). *Significantly different from before the distraction; *p* < .05. *n* = 12.

After the 40-s distraction, SVH decreased by 3.9° in the post-2 phase (i.e., 30–80 s after the distraction) of the 1.8-G trial [*F*(2, 11) = 5.35, *p *= .02], but it was not altered in the other trials [1.1-G trial: +3.5°, *F*(2, 11) = 1.95, *p *= .18; 2.5-G trial: +1.2°, *F*(2, 11) = 3.67, *p* = .07] (Figure 2).

### Capacity to Indicate an Imagined Bank Angle at 1 g

**Figure 3.**
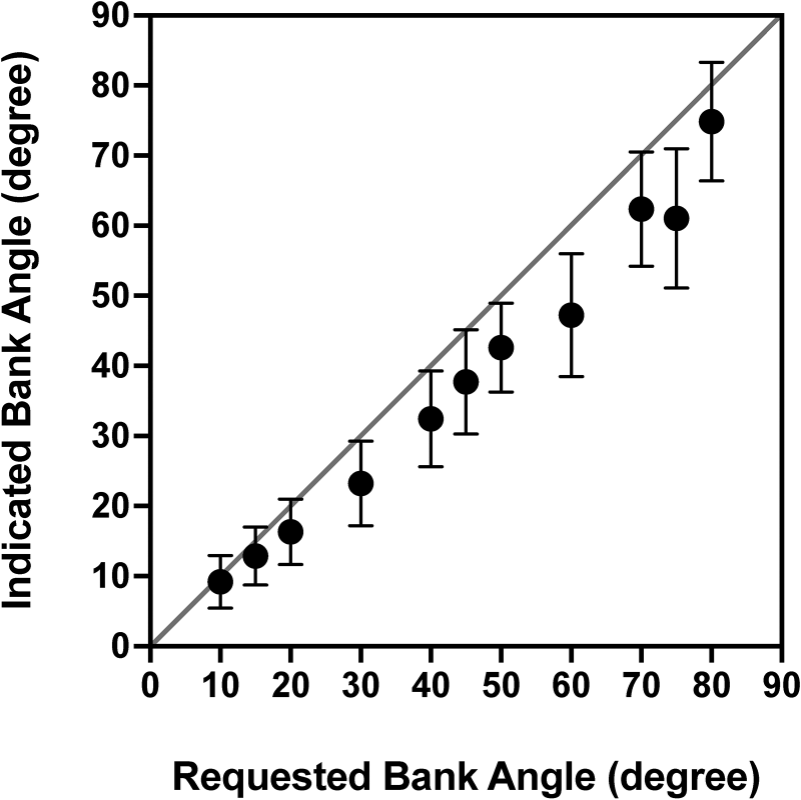
Indicated bank angles (roll tilt angles) as a function of requested bank angles [mean (standard deviation); *n* = 12]. The pilots adjusted the luminous line in the static 1-g environment. Data points are averages of response to the right and left of the vertical. The line of identity represents ideal responses. There was a general tendency on underreporting, i.e., the line was adjusted to tilts smaller than numerically presented.

Generally, the participants’ indications were ∼15.5% smaller (i.e., underreported) than requested (Figure 3). The relative deviation was greater when participants were asked to adjust the bank angle at oblique [i.e., from 15–75°; −17.3 (14)%] than at near cardinal [i.e., at 10° and 80°; −7.2 (21)%] orientations [*t*(11) = 2.20, *p* = .01].

### Knowledge of the Relationship Between Bank Angle and +Gz Load

**Figure 4.**
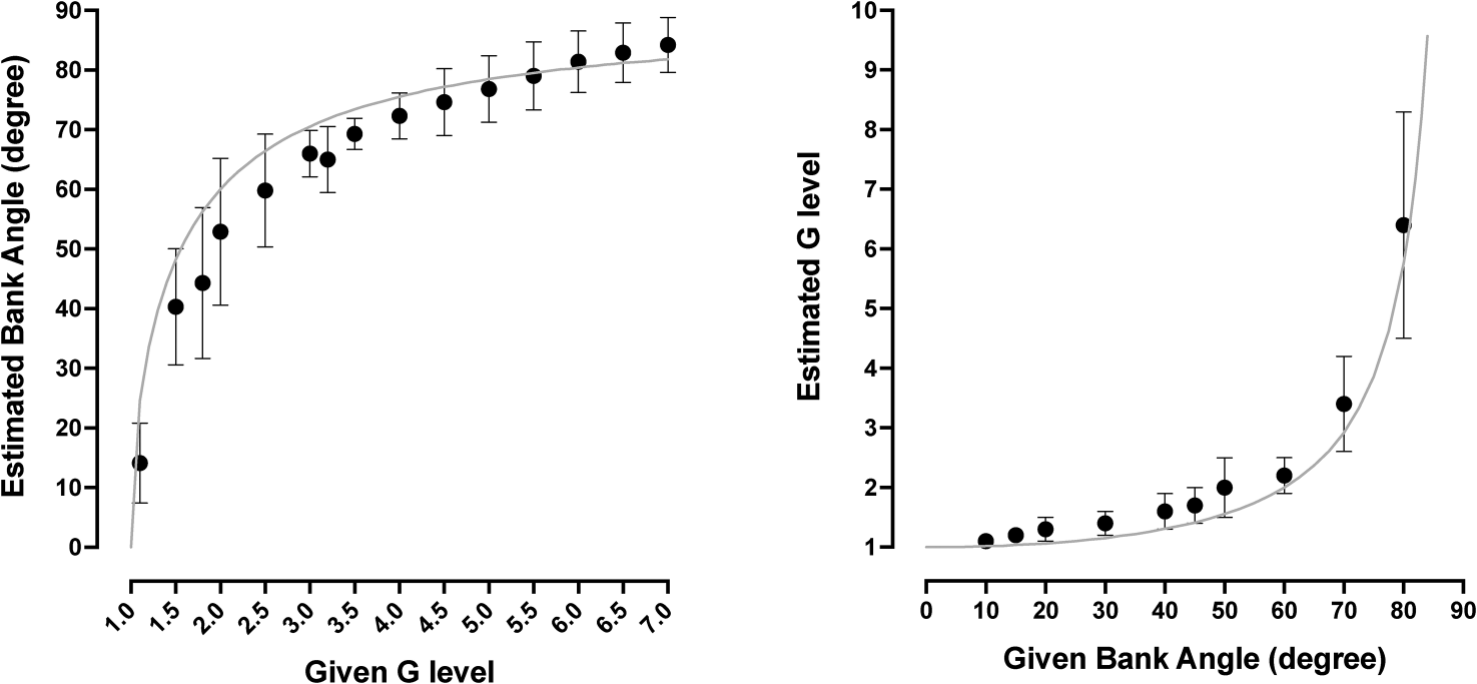
The pilots’ knowledge of the relationship between G level and bank angle. The left diagram shows bank angle responses [data point represents mean (standard deviation), *n* = 12] as a function of the G level. During this task, G levels (range: 1.1–7.0 G) were mentioned by the experimenter; the pilots responded by telling what he believed to be the corresponding bank angle. The right diagram shows, conversely, the pilots’ G level responses as a function of bank angle mentioned by the experimenter (range of given bank angle: 10°–80°). Gray lines represent the true relationship between G level and bank angle.

For any given G load and bank angle, participants, on average, underestimated the bank angle by ∼4% and overestimated the G load by ∼17%, respectively (Figure 4). When the G load was given and the bank angle was requested, the interindividual response variance described by the differences in the size of standard deviation was larger (*F* = 4.92; *p* < .001) at low (i.e., 1.1–2 G; mean standard deviation: 17.9°) than at moderate-to-high (i.e., 2.5–7 G; mean standard deviation: 8.1°) G loads. When the bank angle was given and the G load requested, the response variance was larger (*F* = 151.66; *p* < .001) for moderate-to-high (i.e., 30°–80°; mean standard deviation: 1.8 G) than for low (i.e., 10°–20°; mean standard deviation: 0.1 G) bank angles.

### Correlations

**Figure 5.**
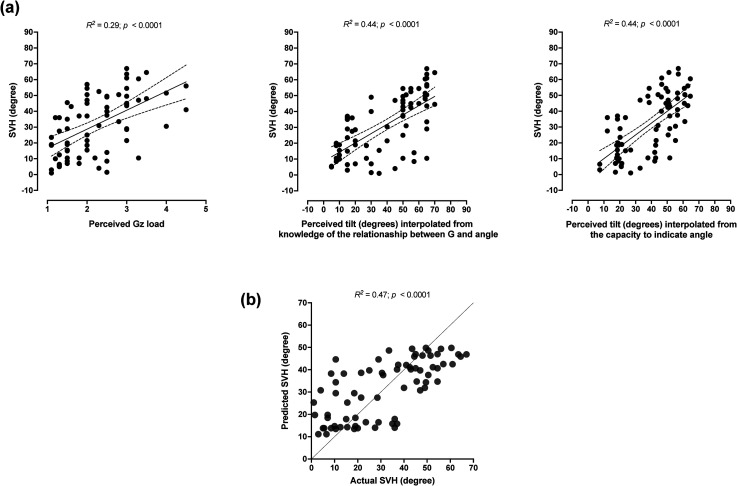
(a) Pearson's product–moment correlation between the subjective visual horizontal (SVH; the mean of initial and final SVH values obtained in each trial/session) and the +Gz load perception (i.e., the G value provided by each participant in the corresponding trial/session), the perceived tilt interpolated from knowledge of the relationship between G load and bank angle [i.e., the interpolated values derived from the individual data obtained in subtask 1, wherein the experimenter numerically mentioned G levels (range: 1.1–7.0 G) and the participant responded verbally with corresponding bank angles] and the perceived tilt interpolated from the capacity to adjust the angle in a 1-g environment [i.e., the interpolated values derived from the individual data obtained in the task, wherein the participant was requested to, by using the luminous line, indicate 22 numerically presented angles ranging from 10° to 80°, while he was at 1 g]. The solid line represents best fit by linear regression, and the dashed line indicates 95% confidence interval (b) Actual and predicted SVH values derived from the multiple regression model.

SVH was moderately correlated with the perception of G load (*r* = .54) and largely correlated with the knowledge of the relation between Gz load and bank angle (*r* = .66) and the capacity to adjust the line according to the numerically given values (*r* = .67) (*p* < .001; Figure 5a).

### Relative Contributions to SVH

The multiple linear regression model was significant (*p* < .001; Figure 5b). The knowledge of the relationship between bank angle and G load (40%; β = 0.30, *p* = .05) and the capacity to adjust the line to the requested angle (55%; β = 0.41, *p* = .05) appeared to constitute the main contributors of the model, whereas the perception of G load did not add significantly to it (β = −0.03, *p* = .99).

## Discussion

Present results demonstrated that, on average, the pilots underestimated their roll tilt, initially and after 3–4 min of the elevated G exposure, with no differences between initial and final SVH values at the 1.1 and 1.8-G loads, but with a slightly increasing SVH during the course of the second 2.5-G plateau. The average SVH was 4°–28° less than the true roll angle at the different G loads and times at the G plateaus, but the SVH settings exhibited large interindividual differences. At all G loads, SVH was similar in the first and second sessions. The visuospatial distraction task did not affect the SVH settings at 1.1 or 2.5 G, nor initially at 1.8 G, whereas the second postdistraction value at 1.8 G was 4° lower than the predistraction value. Further, the multiple regression analysis indicated that the chain of tested capacities—perception of G load, knowledge of relation between G load and bank angle, and accuracy when indicating angles at 1 g—significantly contributed to the SVH.

### Reliability and Distractibility of SVH

Despite the long period elapsing between the two experimental sessions, there was very little variation between the first and second sessions as regards initial, final, and overall (trial average) SVH. This is in good agreement with previous findings in pilots (Tribukait et al., 2011), pilot students (Tribukait et al., 2013), and naïve participants (Tribukait et al., 2013), and confirms the notion that SVH constitutes a highly reliable variable during exposures to increased G load in a centrifuge. It should be noted, however, that in our previous experiments, the participants/pilots have always been instructed to indicate their spontaneous imagination of the horizon and to avoid any conscious “calculation” of the roll position (Tribukait et al., 2011, 2013, 2016), whereas in the present experiments, the pilots were, by contrast, encouraged to use all available cues and capacities when setting the luminous line parallel with the horizon. Notwithstanding, present findings were similar to those obtained in the previous study, with the present initial SVH averaging 32° and 42° in the 1.8-G (56° inclination) and 2.5-G (66° inclination) runs, respectively, as compared to an initial 31° SVH indicated in a 2-G exposure (60° inclination) by fighter pilots instructed to merely indicate their spontaneous imagination of the horizon (Tribukait et al., 2011, 2023). Even though these three studies were performed in different pilots, and hence their outcomes should be compared with caution, it appears that the present pilot group did not benefit substantially from using all available cues and experience when estimating the roll inclination. However, in both the present and previous studies, there was a large interindividual range of the initial SVH responses, with a few pilots indicating nearly the correct roll inclination whereas others indicating virtually no perceived tilt. Such interindividual variability in SVH response at increased G load, ranging ∼70° in the present 2.5-G trial, is remarkable even when accounting for a substantial interindividual variability (0.6°–2.1°) among healthy individuals as regards their roll tilt vestibular perceptional direction recognition threshold at different G loads (Rosenberg et al., 2018).

Both in a previous study at 2.0 G (Tribukait et al., 2011) and in the present study at the 1.1- and 1.8-G loads, the majority of the pilots maintained their initial perception throughout the exposure (Table 1), which is at variance with naïve participants (nonpilots) in whom the initial SVH is typically considerably smaller and declines exponentially with a time constant of 1–2 min (Tribukait & Eiken, 2016). Such a response is attributable to the transient semicircular canal stimuli, induced by the planetary acceleration of the centrifuge and swing-out of the gondola, with a combination of canal stimuli in the transversal (yaw), frontal (roll), and sagittal (pitch) planes (Tribukait & Eiken, 2005b). The sensation of roll-angular displacement results from velocity-to-position integration and storage of the displacement information, presumably with involvement of the hippocampus and the head-direction system; the decay of the SVH tilt would represent the forgetting of the initial displacement information (Tribukait & Eiken, 2016). It can be assumed that also in pilots, the forgetting of the initial vestibular sensation of roll tilt follows an exponential curve with a time constant of 1–2 min. Notwithstanding, at the 1.1- and 1.8-G loads, the pilots maintained the initial SVH setting throughout each G trial, and during the course of the second 2.5 G trial, the pilots even increased the SVH setting. One might then consider whether the nondecaying SVH simply reflects a short-term visual memory, consolidated by the iterative settings of the luminous line. However, the visual working memory is sensitive to distraction. In particular, forgetting is more pronounced if there is, as in the present situation, a featural congruence between the memory target and the distractor, i.e., if the two tasks are of similar character but not identical (Baddeley, 2003; Lorenc et al., 2021; Magnussen et al., 1991; Oberauer et al., 2018). Therefore, the persistence of SVH in the pre- vs. postdistraction settings (Figure 2) suggests that settings were dependent on other mechanisms than visual working memory.

Judging by the increasing SVH during the course of the second 2.5-G plateau, it appears that, at this G level, the impression of roll tilt increased over time. The mechanisms behind such reinforcement of roll tilt perception at high G are not clear. It is well established that under steady-state conditions at increased +Gz load, a head tilt creates an exaggerated impression of head angular displacement, commonly termed “G-excess illusion,” because the G force-induced excess stimulation of the otoliths potentiates the displacement information provided by the semicircular canals (Clark et al., 2015; Miller & Graybiel, 1966; Schoene, 1964). In the present experiments, by contrast, the G vector was always aligned with the head-and-body long axis, and hence, regardless of G load, the otoliths were always signaling vertical body posture. Consequently, upon initiation of a high G exposure in the centrifuge under such conditions, the detection of roll-angular displacement from semicircular canal input is blunted by opposing input from the otoliths, signaling preserved upright posture (Tribukait & Eiken, 2005b; Young, 1984). It is therefore noteworthy, that in nonpilots instructed to act intuitively, the gradual decline of the SVH during the subsequent G plateau is slower at markedly than modestly elevated G (Tribukait & Eiken, 2006). Thus, a continual high graviceptive stimulus in the head-to-seat direction seems to entertain the memory of the initial roll displacement in nonpilots (Tribukait & Eiken, 2006), and to reinforce the sensation of roll tilt in pilots.

### Perception of G Load

During the initial phase of every 6-min centrifugation, each pilot was asked to assess the G load. Presumably by conscious and subconscious integration of input from different somatosensors, i.e., tactile (e.g., increased pressure down the seat) and proprioceptive (e.g., enhanced muscle tension) (Young, 1984), all pilots were able to rank the three G loads. Yet, and despite their flying experience, the pilots’ assessments of G load were generally not accurate, with an average overestimation of 20%–30% for all three G levels. It appears that the majority of studies concerning subjective assessments of weight or force concur with similar overestimation, irrespective of the experimental paradigm. For instance, in a 1-g environment, healthy individuals typically overestimate the weight of a hand-held object by 15%–20% (Engen, 1988; Warren & Warren, 1956), and in hypergravity (e.g., during parabolic flight experiments), participants have been reported to substantially overestimate the G-induced increase in weight of the entire body and parts of the body (Ferre et al., 2019; Lackner & Graybiel, 1984). The mechanisms underlying such overestimation of weight/force field remain to be investigated.

A central question is to what degree feedback or training procedures will affect the accuracy of the force field estimates in hypergravity. Gracio et al. (2009) repeatedly exposed participants without any previous high G experience to two different G loads (1.6 G and 1.8 G) in a centrifuge. The participants rapidly improved their capacity to actively adjust the G load, which might suggest a rapid perceptual learning (Gracio et al., 2009). Still, the present group of pilots, all well accustomed to high G loads, verbally overestimated the force levels, and their magnitudes of overestimation were consistent in the two experimental sessions, occurring with a 6-month interval. Moreover, present results appear to be in line with our previous study, in which fighter pilots exposed to a 6-min 2-G turn overestimated the G load while underestimating the roll tilt (Tribukait et al., 2011). Based on this, we reasoned that the sensation of roll tilt was not dependent on conscious awareness of the G environment and therefore likely not reflecting an intellectual process (Tribukait et al., 2011). Present results not only confirm the notion that fighter pilots typically are inaccurate in their G assessments but also extend it to include situations where they are not acting intuitively but requested to use all available cues when estimating the load. Nevertheless, present analyses indicate that, under such conditions, the pilot's ability to perceive the G load may play a role for correct assessment of roll-angular displacement during sustained centrifugation. It should be noted, however, that the correlation between perception of G load and SVH was merely moderate, and perception of G load did not significantly add to the predictive value of the multiple regression model. The relatively narrow range of G loads in the present investigation (1.1–2.5 G corresponding to roll angles 25°–66°) may have limited the impact of G perception in the model; thus, the other two abilities included as independent variables in the prediction model were investigated across a wider range of angles (10°–80°).

Moreover, the pilots maintained a constant SVH throughout each 1.1- and 1.8-G exposure and increased the SVH setting during the course of the second 2.5-G exposure. It appears plausible that the G-induced somatosensory input contributed to the pilot's stable or increasing SVH settings during prolonged centrifugation. Thus, virtually all pilots were also able to deduce that, in all three trials, the G load was maintained constant. They reported that the bodily sensation of constant and elevated G load was consistent with performing a prolonged coordinated level turn. In fact, they could not fathom any alternative flight maneuver producing such sensation. Yet, as mentioned, the increasing SVH values during the course of the second 2.5-G exposure might suggest that they sensed a gradual “tightening” of the turn in this condition.

### Indication of Angle

In the current study, we also assessed the capacity of the pilots to set the luminous line (without visual cues), at a number of requested angles (ranging from 10° to 80°), while seated upright in a static, 1-g environment. On average, the set angles deviated from the true (requested) angles by approximately −15%. Although the errors differed considerable between individuals, there was a general tendency of the indicated tilts being smaller than the requested. It is noteworthy, however, that the errors were more prominent at oblique than at near horizontal or vertical angles (Figure 3). Two phenomena may have contributed to this deviation, namely, the “oblique effect” and the “horizontal–vertical illusion.” The oblique effect, which has been confirmed in numerous studies (Jastrow, 1893; Lipshits & McIntyre, 1999; McIntyre et al., 2001), implies that, in humans, as well as in other species, the representation of visual orientation is more accurate for cardinal than oblique orientations (Appelle, 1972; Girshick et al., 2011). The effect seems to originate from within the visual cortex, but its neural pathways are unclear (for review, see Li et al., 2003). Thus, the oblique effect might explain our finding that the settings were more accurate for the near horizontal and vertical than oblique angles but cannot explain the direction bias of the errors at oblique angles. Presumably, the horizontal–vertical illusion, which signifies the tendency to overestimate the length of vertical compared to horizontal lines, played a role (Avery & Day, 1969; Hamburger & Hansen, 2010). Thus, when the pilots attempted to set the luminous line at a given oblique angle, they may, in an imaginary coordinate system, have overestimated the vertical component of the line and erroneously compensate for that by a deviation toward the horizon. Considering that neither the oblique effect nor the horizontal–vertical illusion seems to be affected by changes in the gravitoinertial force field (based on trials performed in microgravity) (Lipshits et al., 2005; McIntyre et al., 2001), it is possible that the pilots’ tendency to underindicate oblique angles may have contributed to their underestimations of the roll tilt of the gondola. This notion is also supported by the finding in the multiple regression analysis that the capacity to indicate numerically presented angles contributed to the ability to estimate roll tilt.

### Knowledge of Relation Between G Load and Bank Angle

The pilots’ conceptual understanding of the relation between G level and bank angle—or their specific knowledge of how a number of G levels, frequently encountered during flight, correspond to bank angles, or vice versa—showed considerable deviations from the mathematical truth. During a normal level aircraft turn, i.e., conducted at constant altitude and air speed, the G load is inversely related to the cosine of the aircraft bank angle (G force = 1 g/cos bank angle); an analogous relation exists in the centrifuge (G force = 1 g/cos gondola inclination). Even though this fundamental relationship should be well known to the pilots already after their basic flight training and in spite of the fact that they would have had numerous opportunities to experience this relationship during flight, e.g., by linking instrument indications for bank angle and G level or via associating the bodily experiences of G load with visual impressions of the Earth horizon, their capacity to convert given G loads to bank angles was, with few exceptions, poor. Presumably, the generally poor and highly variable knowledge of the G load–bank angle relationship is largely attributable to its marked nonlinearity, rendering the knowledge rather abstract and counterintuitive (Chappell, 2012).

Although no direct comparison was made since there was not a complete correspondence between given G loads and given bank angles in the two knowledge subtasks, it appeared that the order in which the G load–bank angle translation was conducted might have affected the outcome, with seemingly larger errors occurring when the pilots translated a given G load to an angle than vice versa. It remains to be settled if such orderly discrepancy might be related to the pilot's flight experience, with the bank angle being his primary concern during a coordinated turn and the G load representing the secondary/dependent variable.

**Figure 6.**
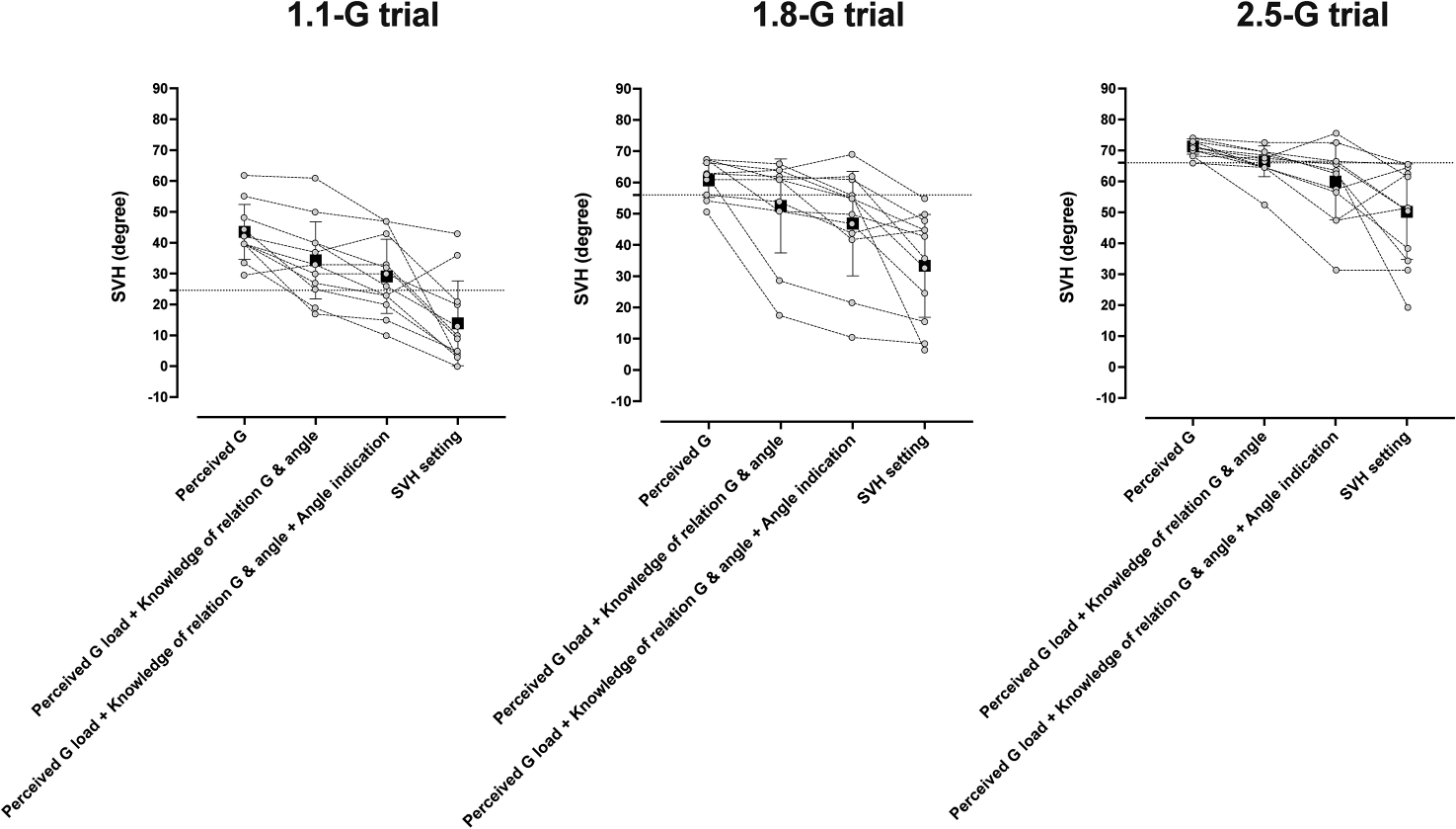
Illustration of the expected and actual SVH settings at the 1.1, 1.8, and 2.5-G loads. The first three columns, from left to right, depict expected SVH setting based on (a) perceived G load converted to true roll angle, (b) perceived load and knowledge of relation between load and angle, and (c) perceived load, knowledge of relation between load and angle, and ability to indicate angle. Column 4 depicts actual SVH settings. Open circles are individual values, retrieved by interpolation, and filled squares mean values. *n* = 12. Note that since the pilots overestimated the G loads, then where perceived load the sole determinant of the SVH setting, an overestimation of the SVH would be expected (first column from the left, at each G trial). When also taking into account that the pilots typically underestimated the angles corresponding to these loads, then the expected SVH value would be lower (second column from the left at each G trial). When also taking into account that the pilots indicated smaller than intended angles, then the expected SVH value would be even lower (third column from the left at each G trial). Yet, the pilots’ actual SVH settings (column furthest to the right at each G trial) were considerably lower than expected based on all three aforementioned abilities (third column from left at each G trial).

Regardless, in the present experiments, the ability to indicate the roll inclination (i.e., SVH) might rather have depended on the capacities to correctly sense the G load, translate it to an angle, and indicate the angle. Even though the present pilots were, on average, mediocre with respect to all three capacities, the multiple linear regression modeling revealed that these capacities combined were significantly related to the pilot's perception of the gondola roll inclination. Therefore, and because a few of the pilots were indeed quite skilled (accurate) in all three capacities (Figure 6), it appears that certain pilots may be able to correctly perceive the G load, consciously estimate, and accurately indicate the corresponding bank angle. Consequently, it may also be warranted to establish whether, and to what extent, it is possible to, by specific training of these capacities, improve the ability to determine the roll inclination when performing a coordinated turn without visual cues. Since it appears that the sensation of bank angle during a level flight turn is virtually identical to the sensation of roll tilt during constant load-centrifugation (Tribukait et al., 2023; Tribukait & Eiken, 2016), such training might be conducted in a centrifuge and any acquired skills then transferred to real flight conditions. Such training regimens, might, however, be challenging, not only because of the nonlinear relation between G load and roll angle, resulting in prominent roll tilt even at minute changes in load, but also because of the brain's inherent reliance on otolith information for spatial orientation. Thus, it was striking in the present study that the pilots’ actual SVH settings at a given load were generally substantially smaller than expected, taking into account their perception of G load, capacity to convert load to angle, and ability to indicate angle (Figure 6). Presumably, this reflects the pilots’ difficulty to, even when convinced of being in a level turn, completely suppress the opposing information from the otoliths of preserved vertical posture.

## References

[bibr1-03010066231209847] Allred A. R. Clark T. K. (2023). Vestibular perceptual thresholds for rotation about the yaw, roll, and pitch axes. Experimental Brain Research, 241, 1101–1115. 10.1007/s00221-023-06570-4 36871088

[bibr2-03010066231209847] Appelle S. (1972). Perception and discrimination as a function of stimulus orientation: The “oblique effect” in man and animals. Psychological Bulletin, 78, 266–278. 10.1037/h0033117 4562947

[bibr3-03010066231209847] Avery G. C. Day R. H. (1969). Basis of the horizontal–vertical illusion. Journal of Experimental Psychology, 81, 376–380. 10.1037/h0027737 5811814

[bibr4-03010066231209847] Baddeley A. (2003). Working memory: Looking back and looking forward. Nature Reviews Neuroscience, 4, 829–839. 10.1038/nrn1201 14523382

[bibr5-03010066231209847] Chappell T. (2012). Varieties of knowledge in Plato and Aristotle. Topoi, 31, 175–190. 10.1007/s11245-012-9125-z

[bibr6-03010066231209847] Clark T. K. Newman M. C. Oman C. M. Merfeld D. M. Young L. R. (2015). Human perceptual overestimation of whole body roll tilt in hypergravity. Journal of Neurophysiology, 113, 2062–2077. 10.1152/jn.00095.2014 25540216 PMC4416546

[bibr7-03010066231209847] Engen T. (1988). Psychophysics. States of Brain and Mind, 37, 455–467.

[bibr8-03010066231209847] Ferre E. R. Frett T. Haggard P. Longo M. R. (2019). A gravitational contribution to perceived body weight. Scientific Reports, 9, 11448. 10.1038/s41598-019-47663-x 31391471 PMC6685953

[bibr9-03010066231209847] Gibb R. Ercoline B. Scharff L. (2011). Spatial disorientation: Decades of pilot fatalities. Aviation, Space, and Environmental Medicine, 82, 717–724. 10.3357/ASEM.3048.2011 21748911

[bibr10-03010066231209847] Girshick A. R. Landy M. S. Simoncelli E. P. (2011). Cardinal rules: Visual orientation perception reflects knowledge of environmental statistics. Nature Neuroscience, 14, 926–932. 10.1038/nn.2831 21642976 PMC3125404

[bibr11-03010066231209847] Glasauer S. (1992). Interaction of semicircular canals and otoliths in the processing structure of the subjective zenith. Annals of the New York Academy of Sciences, 656, 847–849. 10.1111/j.1749-6632.1992.tb25272.x 1599198

[bibr12-03010066231209847] Gracio B. J. C. Wentink M. Groen E. Bles W. (2009, August 10–13). Subjective estimates of G-load in centrifuge-based simulation and applications for G-cueing in Desdemona [Paper presentation]. AIAA Modeling and Simulation Technologies Conference, Chicago, IL, United States (pp. 1–8).

[bibr13-03010066231209847] Hamburger K. Hansen T. (2010). Analysis of individual variations in the classical horizontal–vertical illusion. Attention, Perception & Psychophysics, 72, 1045–1052. 10.3758/APP.72.4.1045 20436199

[bibr14-03010066231209847] Jastrow J. (1893). The section of psychology. In Hardy M. P. (Ed.), Official Catalogue—World’s Columbian Exposition (pt. vii, pp. 50–60). W. B. Conkey.

[bibr15-03010066231209847] Lackner J. R. Graybiel A. (1984). Perception of body weight and body mass at twice earth-gravity acceleration levels. Brain, 107, 133–144. 10.1093/brain/107.1.133 6697150

[bibr16-03010066231209847] Li B. Peterson M. R. Freeman R. D. (2003). Oblique effect: A neural basis in the visual cortex. Journal of Neurophysiology, 90, 204–217. 10.1152/jn.00954.2002 12611956

[bibr17-03010066231209847] Lim K. Karmali F. Nicoucar K. Merfeld D. M. (2017). Perceptual precision of passive body tilt is consistent with statistically optimal cue integration. Journal of Neurophysiology, 117, 2037–2052. 10.1152/jn.00073.2016 28179477 PMC5434481

[bibr18-03010066231209847] Lipshits M. Bengoetxea A. Cheron G. McIntyre J. (2005). Two reference frames for visual perception in two gravity conditions. Perception, 34, 545–555. 10.1068/p5358 15991691

[bibr19-03010066231209847] Lipshits M. McIntyre J. (1999). Gravity affects the preferred vertical and horizontal in visual perception of orientation. Neuroreport, 10, 1085–1089. 10.1097/00001756-199904060-00033 10321488

[bibr20-03010066231209847] Lorenc E. S. Mallett R. Lewis-Peacock J. A. (2021). Distraction in visual working memory: Resistance is not futile. Trends in Cognitive Sciences, 25, 228–239. 10.1016/j.tics.2020.12.004 33397602 PMC7878345

[bibr21-03010066231209847] Magnussen S. Greenlee M. W. Asplund R. Dyrnes S. (1991). Stimulus-specific mechanisms of visual short-term memory. Vision Research, 31, 1213–1219. 10.1016/0042-6989(91)90046-8 1891813

[bibr22-03010066231209847] McIntyre J. Lipshits M. Zaoui M. Berthoz A. Gurfinkel V. (2001). Internal reference frames for representation and storage of visual information: The role of gravity. Acta Astronautica, 49, 111–121. 10.1016/S0094-5765(01)00087-X 11669099

[bibr23-03010066231209847] Miller E. F. Graybiel A. (1966). Magnitude of gravitoinertial force, an independent variable in egocentric visual localization of the horizontal. Journal of Experimental Psychology, 71, 452–460. 10.1037/h0022954 5908830

[bibr24-03010066231209847] Oberauer K. Lewandowsky S. Awh E. Brown G. D. A. Conway A. Cowan N. Donkin C. Farrell S. Hitch G. J. Hurlstone M. J. Ma W. J. Morey C. C. Nee D. E. Schweppe J. Vergauwe E. Ward G. (2018). Benchmarks for models of short-term and working memory. Psychological Bulletin, 144, 885–958. 10.1037/bul0000153 30148379

[bibr25-03010066231209847] Rosenberg M. J. Galvan-Garza R. C. Clark T. K. Sherwood D. P. Young L. R. Karmali F. (2018). Human manual control precision depends on vestibular sensory precision and gravitational magnitude. Journal of Neurophysiology, 120, 3187–3197. 10.1152/jn.00565.2018 30379610 PMC6442919

[bibr26-03010066231209847] Schoene H. (1964). On the role of gravity in human spatial orientation. Aerospace Medicine, 35, 764–772. 14215796

[bibr27-03010066231209847] Tribukait A. Bergsten E. Brink A. Eiken O. (2023). Visual measures of perceived roll tilt in pilots during coordinated flight and gondola centrifugation. Journal of Vestibular Research, 33, 1–19. 10.3233/VES-220016 36442173 PMC9986699

[bibr28-03010066231209847] Tribukait A. Eiken O. (2006). Roll-tilt perception during gondola centrifugation: Influence of steady-state acceleration (G) level. Aviation, Space, and Environmental Medicine, 77, 695–703. 16856353

[bibr29-03010066231209847] Tribukait A. Eiken O. (2016). On the time course of short-term forgetting: A human experimental model for the sense of balance. Cognitive Neurodynamics, 10, 7–22. 10.1007/s11571-015-9362-0 26834858 PMC4722133

[bibr30-03010066231209847] Tribukait A. Eiken O. Lemming D. Levin B. (2013). Use of an adjustable hand plate in studying the perceived horizontal plane during simulated flight. Aviation, Space, and Environmental Medicine, 84, 739–745. 10.3357/ASEM.3470.2013 23855072

[bibr31-03010066231209847] Tribukait A. Eiken O. (2005a). Perception of the head transversal plane and the subjective horizontal during gondola centrifugation. Perception & Psychophysics, 67, 369–382. 10.3758/BF03193318 16119388

[bibr32-03010066231209847] Tribukait A. Eiken O. (2005b). Semicircular canal contribution to the perception of roll tilt during gondola centrifugation. Aviation, Space, Environmental Medicine, 76, 940–946. 16235877

[bibr33-03010066231209847] Tribukait A. Gronkvist M. Eiken O. (2011). The perception of roll tilt in pilots during a simulated coordinated turn in a gondola centrifuge. Aviation, Space, and Environmental Medicine, 82, 523–530. 10.3357/ASEM.2898.2011 21614866

[bibr34-03010066231209847] Tribukait A. Strom A. Bergsten E. Eiken O. (2016). Vestibular stimulus and perceived roll tilt during coordinated turns in aircraft and gondola centrifuge. Aerospace Medicine and Human Performance, 87, 454–463. 10.3357/AMHP.4491.2016 27099084

[bibr35-03010066231209847] Warren R. M. Warren R. P. (1956). Effect of the relative volume of standard and comparison-object on half-heaviness judgments. The American Journal of Psychology, 69, 640–643. 10.2307/1419087 13403004

[bibr36-03010066231209847] Young L. (1984). Perception of the body in space: Mechanisms. In Geiger S. (Ed.), Handbook of physiology. Vol III (pp. 1023–1066) American Physiological Society: Bethesda.

